# How do health system factors (funding and performance) impact on access to healthcare for populations experiencing homelessness: a realist evaluation

**DOI:** 10.1186/s12939-023-02029-8

**Published:** 2023-10-17

**Authors:** Rikke Siersbaek, John Ford, Clíona Ní Cheallaigh, Steve Thomas, Sara Burke

**Affiliations:** 1 Discipline of Clinical Medicine, School of Medicine, Trinity College Dublin Trinity Centre for Health Sciences, St James’s Hospital, Dublin 8, Ireland; 2 Wolfson Institute for Population Health, Queen Mary University, Charterhouse Square, London, EC1M 6BQ UK; 3 Centre for Health Policy and Management, Trinity College Dublin, 3-4 Foster Place, Dublin 2, Ireland

**Keywords:** Health services accessibility, Social exclusion, Homelessness, Health services administration, Healthcare financing, Organizational objectives, Realist evaluation

## Abstract

**Background:**

People experiencing long-term homelessness face significant difficulties accessing appropriate healthcare at the right time and place. This study explores how and why healthcare performance management and funding arrangements contribute to healthcare accessibility or the lack thereof using long-term homeless adults as an example of a population experiencing social exclusion.

**Methods:**

A realist evaluation was undertaken. Thirteen realist interviews were conducted after which data were transcribed, coded, and analysed.

**Results:**

Fourteen CMOCs were created based on analysis of the data collected. These were then consolidated into four higher-level CMOCs. They show that health systems characterised by fragmentation are designed to meet their own needs above the needs of patients, and they rely on practitioners with a special interest and specialised services to fill the gaps in the system. Key contexts identified in the study include: health system fragmentation; health service fragmentation; bio-medical, one problem at a time model; responsive specialised services; unresponsive mainstream services; national strategy; short health system funding cycles; and short-term goals.

**Conclusion:**

When health services are fragmented and complex, the needs of socially excluded populations such as those experiencing homelessness are not met. Health systems focus on their own metrics and rely on separate actors such as independent NGOs to fill gaps when certain people are not accommodated in the mainstream health system. As a result, health systems lack a comprehensive understanding of the needs of all population groups and fail to plan adequately, which maintains fragmentation. Policy makers must set policy and plan health services based on a full understanding of needs of all population groups.

**Supplementary Information:**

The online version contains supplementary material available at 10.1186/s12939-023-02029-8.

## Background

### Homelessness and health

People who experience long-term homelessness often have poorer health outcomes than their housed peers [1, 2] and as a result, they have a need for more frequent and more comprehensive healthcare interventions and at the same time, as a population group, they experience more difficulties in accessing healthcare [1–6]. As observed by Tudor Hart in 1976, the people who need healthcare services the most often are the ones who receive it the least [7]. This observation still holds true and with extreme and tragic effects for people at the sharp end of inequality, as typified by people experiencing homelessness and complex needs.

Homelessness represents an extreme form of socioeconomic deprivation and social exclusion [4, 8]. According to the European Typology of Homelessness and Housing Exclusion (ETHOS), homelessness occurs in four ways:

- Rooflessness (sleeping rough, without any shelter);
- Houselessness (having somewhere to sleep but in a temporary shelter or institution);
- Living in insecure housing (e.g., insecure tenancies, threat of eviction, violence); and.
- Living in inadequate housing (overcrowding, unfit housing, caravans on illegal campsites) [9].

Among the total population experiencing homelessness, a subset of people experience long-term or chronic homelessness which is often associated with ‘tri-morbidity’. Tri-morbidity means the presence of mental ill health, physical ill health, and drug and alcohol misuse, which cause and amplify poor health outcomes and leading to premature ageing and frailty. When people who have frequent healthcare needs are unable to access it, health problems often get worse and more complicated to treat. If care is delayed, when a health need is finally addressed, the care needed is often more complex and intensive and it comes with a greater cost [5, 10–13].

There is significant heterogeneity among numerous subgroups within populations experiencing homelessness, including single adults and families, with a more intense burden of ill-health experienced by chronically homeless adults [3, 14–16]. Populations experiencing chronic homelessness access primary care less often than housed populations and use costly unscheduled acute healthcare at a higher rate than their housed peers [3, 4, 6]. Populations experiencing homelessness also have a much earlier onset of chronic illnesses and multimorbidity than their housed peers [3] along with a higher prevalence of problematic substance use and mental ill health [13]. They have often experienced childhood trauma and it is common to have encountered several adverse childhood experiences (ACEs) such as violence in the home, child neglect, child abuse, parental mental illness, and/or parental substance dependency [2, 17, 18]. As a result, individuals who experience homelessness often live lives marked by multiple and enduring disadvantage which takes a profound toll and often results in premature ageing, disability, or death [19].

Populations experiencing homelessness develop physical frailty and cognitive impairment much sooner than their housed counterparts. A report which measured physical frailty and cognitive impairment in a group 31 of people experiencing longstanding homelessness and complex needs living in supported long-term homeless accommodation in Dublin, estimated their biological age to be 10–20 years older than their physical age [10].

### Healthcare access

Healthcare access is not just the act of arriving at a health clinic or hospital and walking over the threshold. The degree to which a health service is accessible is not merely due to its opening hours or the location of a given clinic. In fact, health systems are complex open systems with a multitude of inputs and outputs interacting to produce intended and unintended outcomes [20], a major goal of which is to provide preventive, curative and rehabilitative care to the people who need it at the right time and at the right place. To be able to disentangle how complex high level health system factors impact healthcare accessibility, realist evaluation is a useful approach because it works to understand and explain complexity rather than to try to isolate effects away from it [21].

Based on the work of Aday and Andersen, Penchansky and Thomas, and Levesque et al., we view healthcare access broadly as a process that takes place on a continuum. In successfully accessing healthcare, an individual has to be able to conceive of a health need, feel empowered to act, reach a service, and engage on an ongoing basis to access care continually as needed [22–24].

### Underpinning theory

There is a large body of international research which examines healthcare access for populations experiencing homelessness from the point of view of the individual person who has a health need [1, 11, 19, 25–30]. Studies have identified barriers to accessing healthcare related to:

- **Resources** such as access to means of transportation, lack of childcare, difficulty taking time off work;
- **Knowledge** such as understanding of symptoms of illness and the degree to which healthcare interventions can make matters better, awareness of clinic locations;
- **Psycho-social factors** such as lack of trust in healthcare providers based on previous poor experiences, fear of health interventions, fear of bad news, fear of a bad outcome;
- **Competing life needs** such as making money, finding somewhere to sleep e.g. a hostel bed, acquiring substances;

Such research illuminates important factors that inform policy makers, health system leaders, and practitioners about the barriers that individual people face before arriving at a healthcare service in the first place.

On the other hand, there is a dearth of research examining the healthcare access equation from the health system perspective i.e. those who are responsible for planning, managing, and providing care. Without knowledge to understand how health systems make healthcare more or less accessible for populations experiencing homelessness, there is a risk that the attention is placed inappropriately on individual patients. This inhibits the system finding solutions to problems of inaccessibility but also places responsibility and blame on those people who need services and experience significant vulnerability.

To focus our analysis on high level health system factors which impact healthcare accessibility for populations experiencing homelessness, we have used two frameworks: Aday and Andersen’s ‘A Framework for the Study of Access to Medical Care’ and the WHO building blocks [22, 31].

Aday and Andersen’s framework is divided into five areas: ‘Health policy’, ‘Characteristics of health delivery system’, ‘Characteristics of population at risk’, ‘Utilisation of health services’ and ‘Consumer satisfaction’. Here ‘health policy’ covers financing, education, human resources and organisation, and ‘characteristics of health delivery system’ covers resources (volume and distribution) and organisation (entry and structure), all of which are key to understanding healthcare access from a systems perspective [22]. Meanwhile, the WHO health system building blocks framework conceives of the following inputs into a health system: service delivery; health workforce; information; medical products, vaccines & technologies; financing; and leadership/governance [31].

**Fig. 1:**
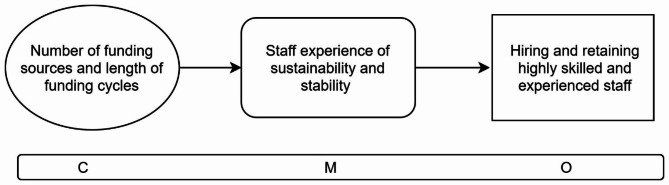
Initial programme theory CMOC1: Funding stability

**Fig. 2:**
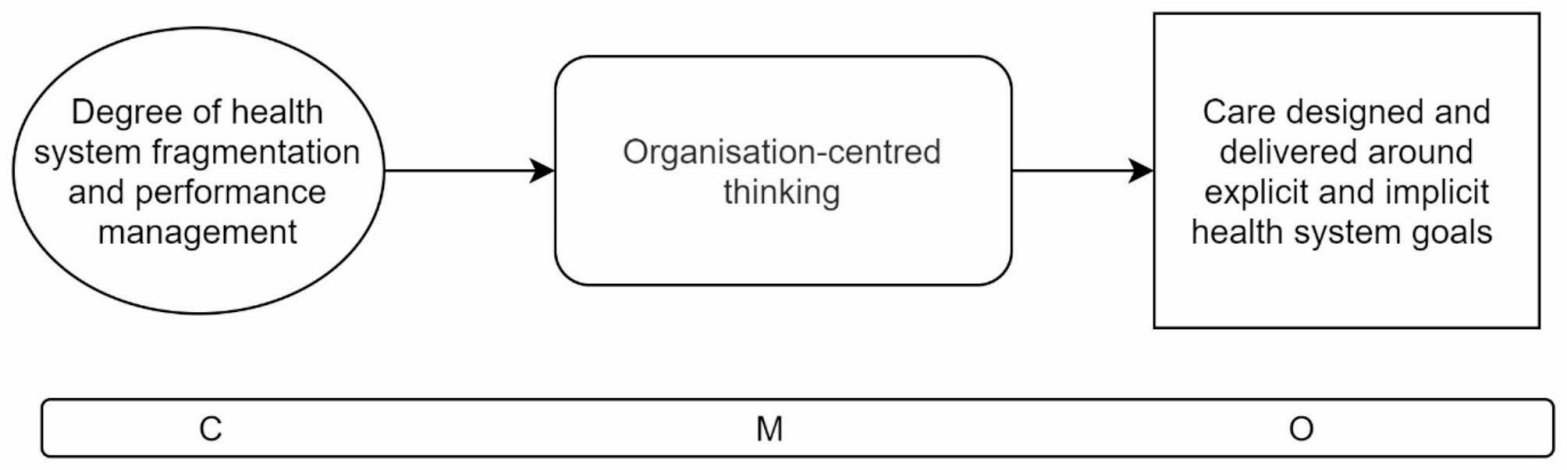
Initial programme theory CMOC2: Health system fragmentation and performance management

The study was guided by initial programme theory developed in our realist review [32]. We identified two Context-Mechanism-Outcome configurations (CMOCs) from the review to focus on (Figs. 1 and 2):

CMOC1 (Fig. 1) suggests that when funding for health services comes from multiple sources in short and unreliable cycles, e.g. grant funding to meet a specific need [13, 27, 33, 34], it leads to a lack sustainability and stability for services [13, 35–38] which face difficulties hiring and retaining skilled and experienced staff members as a result [13, 35, 37–39].

CMOC2 (Fig. 2) suggests that when individual parts of a health system operate with narrowly defined goals in silos [13, 27, 33, 40, 41], performance management measures become focused on meeting those goals and staff prioritise them even in cases where they do not align with the needs of their patients [33, 40, 41]. These performance management practices may inhibit the delivery of services in holistic, coordinated, and flexible ways. As a result, healthcare is organised not around the needs of the person seeking care but around the needs of practitioners and the system [27, 28, 30, 33, 40, 41].

Using these two CMOCs as the starting point, this realist evaluation analyses further the underlying causative relationships they describe between funding procedures and health system performance management, and the ability of service settings, staff, and practitioners to make themselves accessible to populations experiencing homelessness.

## Aim

The aim of this realist evaluation is to understand how funding procedures and health system performance management impact service settings, staff, and practitioners, and their ability to make services accessible to populations experiencing homelessness.

The following research questions were under consideration:

- How do health service funding and performance management impact the accessibility of health services for populations experiencing homelessness?
- For whom and in what circumstances do funding arrangements and performance management work and not work, and why?

## Methods

We undertook a realist evaluation in accordance with an a priori protocol [42]. This theory driven approach aims to identify underlying generative causal mechanisms at play in a complex social programmes which produce a given outcome but only in the right context [43].

A total of twelve people were recruited for the study using purposive sampling and snowball sampling. Interviewees were recruited based on their direct and significant experience providing health and social care services to populations experiencing long-term homelessness or working in the management of such services. Thirteen interviews with twelve interviewees (one person was interviewed twice) provided sufficient data to reach theoretical saturation (see Table 1).

Interviews were guided by a flexible realist interview schedule developed using approaches described in the RAMESES II guidelines and by Manazano et al. [44, 45].

Interviewees had several different professional roles within health services for populations experiencing homelessness with perspectives ranging from physical healthcare, mental healthcare, health service planning, and advocacy.

**Table 1:** Interviewees

Professional	Count
Doctor	3 (one hospital consultant/two GPs)
Nurse	3 (two hospital/ one community)
Senior healthcare manager	2
Social worker	2 (one community/one hospital)
Health service planner	1
Homelessness advocate	1
**Total**	**12**

We undertook realist interviews in a manner whereby the interviewer explains something about the programme theory to the interviewee and seeks information to expand, clarify, reject, and further develop the theorising started in the initial programme theory. The researcher sets out to test theories and to answer questions about the how, for whom, to what extent, and why of the intervention or area under consideration [44]. This was done by ‘telling a story’ about the programme theory without explicitly explaining the theory in realist terms, and then going through a cyclical process where the interviewer and interviewee take turns being the ‘expert’, also known as the ‘teacher-learner cycle’ [44].

The data collected from the 13 interviews were used to create 14 CMOCs, configuring the pertinent findings. Four consolidated CMOCs, presented below, were constructed by combining and merging the findings in the 14 CMOCs to create more generalisable, high level causal explanations. The four CMOCs were the result of iterative cycles of analysis of the primary data collected, with the input of the full research team as well as one-to-one zoom calls with one interviewee and two people with lived experience of homelessness who now work in homeless services. These consultations acted as an approximate, virtual ‘expert panel’ at an advanced stage in the research process which helped to further confirm, challenge, and refine findings from the realist evaluation.

### Coding

Coding was done inductively in the coding style described by Papoutsi et al. (2018, pp. 13–14) and Tierney et al. (2020). First, for the first few interviews, conceptual codes were created inductively as coding took place using NVivo software version 12. Second, those codes were applied subsequent interviews with new codes created along the way if a new concept came up.

Next, CMOCs were crafted to configure the causal relationships observed in the data. For each CMOC, all the data in the form of direct quotes from the interviews were listed in an excel sheet in columns next to the CMOC itself.

## Results

Using the data collected from 13 interviews, we developed fourteen CMOCs. There were further merged into four consolidated CMOCs (see supplementary file). The four consolidated CMOCs are described in detail below.

### Consolidated CMOC 1: Health System Fragmentation

**Fig. 3:**
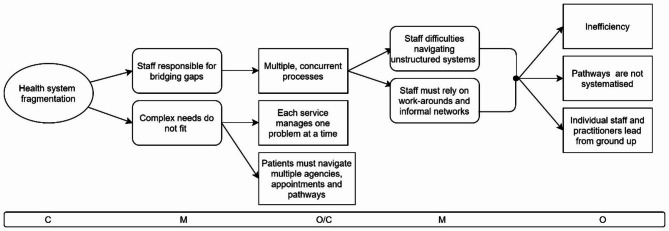
CMOC1 Health system fragmentation

CMOC1 (Fig. 3) explains what happens in the context of providing healthcare services to populations experiencing homelessness in a fragmented manner and in fragmented settings, typically a larger mainstream healthcare organisation like a hospital or within a mainstream health system with multiple settings.

Fragmentation here is both across different systems and services, and within the same system, e.g. across different departments of a hospital, and it creates difficulties for both patients accessing care but also for healthcare practitioners seeking to provide the best care possible for their patients. The following quotes exemplify this CMOC:

I found that by following, you know, the primary care practitioners, public health nurse, GP, the different services by hanging around I actually saw the same person, that’s how I know you know, when I say to the person who looks like seven people to the system I saw the same person coming in, in this domain. And then I was in another domain, the actual same person, and the two services had never spoken, and the three services and then in another domain, so that the problem of that homeless person or the drug user looked far more complex from the fragmented service because it was fragmented. – Interviewee 12.

And

. the biomedical model where it’s one disease that you’re treating doesn’t fit in very well with social exclusion, where people have multiple diseases, multiple things going on, I would actually argue that that one disease at a time fits very few cases that very few people, but but particularly in socially excluded people – Interviewee 1.

In these fragmented, complex contexts, staff and clinicians are responsible for bridging the gaps in a system where interventions are typically intended to resolve one problem at a time with a focus on biomedical health. This approach does not take into account the complexity of health conditions that are often the result of a combination of physical and mental health factors along with addiction behaviours.

The notion that the responsibility for filling gaps is left to individual practitioners came up in several interviews, including this from Interviewee 5:

As much as it like has seen huge amazing, amazing progress in terms of awareness and things like that but, it is very much based on individuals, and those individuals when they’re not there, you know. And I suppose it shouldn’t be like that because that’s how it fails, you know. – Interviewee 5.

A concrete example of bottom-up leadership taking responsibility for improving services due to system fragmentation is a weekly multi-disciplinary meeting of clinicians from hospital and community settings, housing providers, addiction services, the Health Service Executive and more, situated in Dublin city. Started several years ago by a hospital consultant and a manager at an addiction service, it has since grown to over 30 people representing different services. This model of staff convening and coordinating with colleagues was mentioned in several interviews as being useful and it has been replicated within individual service settings.

As a result of frontline staff having to take responsibility for creating healthcare access, patient needs are met in a piecemeal manner and patients must navigate multiple pathways and agencies, and must keep a variety of appointments. Meanwhile, clinicians and staff have to manage multiple concurrent processes on their own inititaitve which is difficult and time-consuming in a system that is not structured to support the navigation of complex needs. Clinicians and staff come up with work arounds and leverage their professional networks to fill the gaps in the systems which leads to the system being inefficient with pathways that are not systematised but forged on a on-to-one basis in each circumstance, which are the responsibility of staff and clinicians to lead and manage from the ground up.

These outcomes were discussed widely in the interviews, as in the following example from Interviewee 6 who talks about the bio-medical model of dealing with one health concern at a time and that patients sometimes do not get their various problems treated because the treatment model is not set up to support the full needs of a person:

But also I suppose the system as it stands is somebody comes in with one particular, some people go to a GP, one medical condition. . they’re coming for a surgical, they’re just dealing with the surgical issue [even though the patient has other concurrent health problems]. And it’s expected because this is what happens in life is that letter will go back out to their GP, they’re attending their outpatient appointment for any other conditions. And what is the problem? Where it isn’t specialised [eg inclusion health service], because you have someone coming in who fracturing their jaw, they haven’t been seen maybe by epilepsy service for so long. Are they engaging with their meds? Are they not? And it’s, he’s someone who’s going to run so I’m going okay, so [name of doctor] can you see this person before they go, a lot of the team particularly medical team will link them in with say hepatitis.’ – Interviewee 6.

### CMOC 2: mainstream and specialised services

**Fig. 4:**
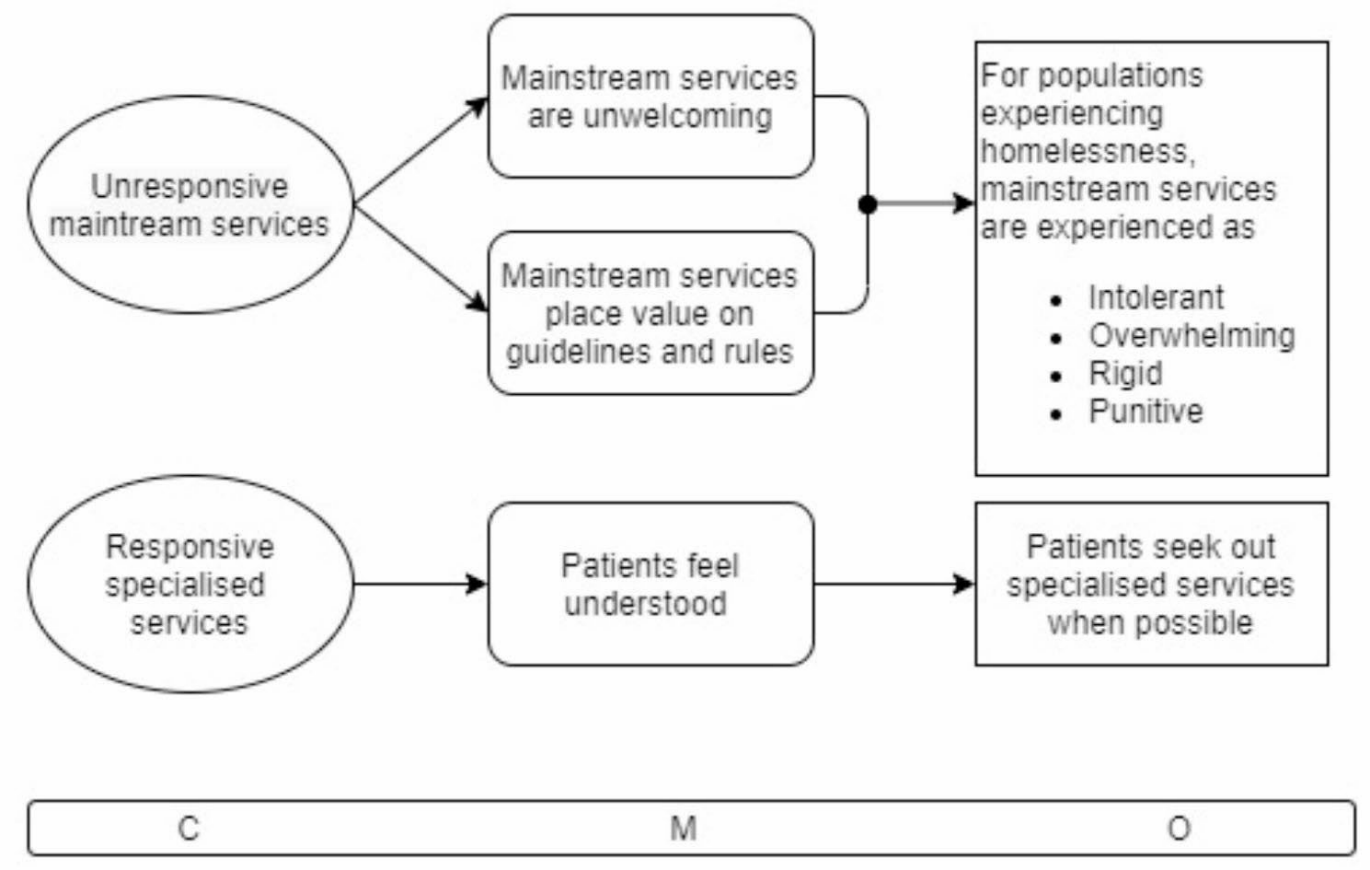
CMOC2 Mainstream and specialised services

CMOC2 (Fig. 4) provides an explanation of the role of specialised homelessness health services in making health systems accessible for populations experiencing homelessness. It shows two sides of the same coin – where mainstream services are unresponsive to the needs of populations experiencing homelessness and specialised services are responsive.

The contexts highlight that typically specialised services for homeless populations are more responsive to their needs than mainstream, non-specialist services. These specialist services can be standalone health practices, e.g. a primary care centre or GP practice that primarily cater to socially excluded populations, a dedicated inclusion health service within a hospital setting, or a clinic within a homeless accommodation setting.

Responsive specialised health services are more likely to employ people with significant exposure to and experience with socially excluded populations who understand their particular needs better than mainstream services. Therefore, when accessing specialised services or when encountering a specially trained staff member in a hospital setting, patients are more likely to be understood and listened to. This in turn means that they are more likely to feel a degree of psychological safety, and that their full set of needs at that moment in their life are understood and will be acted on as much as possible. This leads to the outcome that patients prefer to access specialised services instead of mainstream ones.

At the same time, unresponsive mainstream services are often experienced as unwelcoming by homeless populations. Both staff and patients see mainstream services as being rule-bound and focused on meeting guidelines. Interviewee 4 explained how this happens:

It has cost me a lot of fall out professionally with other clinicians trying to access care for my population because mainstream services follow the guidelines and they follow strict appointment times and well you didn’t show up three times so now you have to go back to the doctor and I’m like, grand, I’m writing the referral right now, no you give them another appointment like you’re just making me go through a process that doesn’t need to happen because somebody said that you needed to do it. – Interviewee 4.

The outcome that follows is that people experiencing homelessness find mainstream health services to be intolerant of their appearance or other characteristics, overwhelming because they are not designed with vulnerable populations in mind and can be difficult to understand and navigate, rigid in the rules and in the narrow scope of behaviour that is accepted, and punitive when one does not understand or is not able to follow the rules.

Regarding the design of mainstream health services Interviewee 2 said:

The mainstream services are not designed for homeless people, they’re designed for housed people. And they suit the needs of housed people. They do appointments. . you keep regular times, people whose behaviours are not chaotic. And anyone who goes into that system who feels out of that system, outside that system, will automatically find it difficult. – Interviewee 2.

Interviewee 6 said the following about the fact that complex needs do not fit in traditional hospital structures:

If we’re trying to get someone up for ages and then the plan is like that I can go and meet them in ED, again it’s a bit hit and miss because there’s only one person [who regularly contacts the Inclusion Health Team]. They tend to ring me a lot about people coming up to ED, there isn’t a fast track way of getting through ED. . you’ve heard many examples of people leaving for things like that. Erm and it’s the way they come across as well, trying to get their needs met, that they just, again, don’t fit in the system. But somewhere, sometimes it does work. – Interviewee 6.

Additionally, the two pathways within CMOC2 indicates the reliance of the mainstream system on specialised services to provide quality care for populations experiencing homelessness. The existence of this parallel system enables the mainstream system to rely on knowing that there is the safetynet of specialised services who will pick up patients who fall through the cracks or are allowed to do so. Interviewee 5 touched on this aspect:

I don’t feel like it’s a health care system I think they’re happy to have people that are doing these system pieces. You know if they can get outcomes, even better, and if we don’t have to finance much, even better. And we can show improved health, great, but if those people aren’t there [the practitioners with a specialist interest in inclusion health] you’d wonder, are the systems there to keep it going? – Interviewee 5.

### CMOC 3: National strategy

**Fig. 5:**
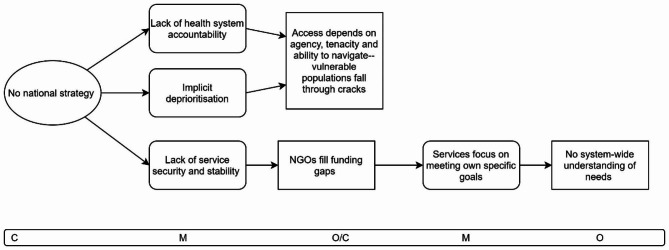
CMOC 3: National strategy

CMOC3 (Fig. 5) explores the importance of national strategic leadership in setting expectations and accountability and shows what happens when there is no specific national strategy for inclusion health, as was the case in Ireland where the data collection took place, March-June 2020. In such a context, there is a lack of explicit accountability for the health system to meet the needs of socially excluded populations and there is an implicit deprioritisation of the area compared with other specialities that have individual national strategies putting them on the policy map. The outcome is that access to health services depends on individual patients and their families’ ability to navigate the systems and use their agency, skills, connections, and tenacity to ensure adequate access. But for population groups who may not have contact with family and/or are not always able to or interested in advocate for themselves in a hostile environment, such as homeless populations, access is impaired.

Interviewee 8 explained:

It’s very challenging for someone that does not see themselves as worth any care, to then be able to engage in a care system as an individual. That is the individual that [misses] an appointment once or twice, and then they’re off the list. .it’s the individual that needs to understand the language that is used by the doctor or the nurse or the caregiver who are looking after them. – Interviewee 8.

The same context, where there is no national strategy for inclusive healthcare, also negatively impacts the stability of sources of funding and dedicated resources coming into the area which means that services do not have permanent and stable resourcing. Interviewee 7 discussed this issue:

The issue here is that we haven’t had secure. . recurring funding. It’s been once off funding. [I]t hasn’t been mainstreamed, which. . create[s] uncertainty for organisations and. . create[s] that instability, in the system. . I think the system is getting a sense, it’s not going away. But it still is once off. – Interviewee 7.

The result is that homeless service NGOs are relied on to observe and prioritise filling the gaps in the mainstream services. Within the mainstream services, individual specialists and specialist teams may also apply for special funding or to hire more staff to be able to provide care in a more holistic, inclusive way. However, providing specialist care from within a large system takes more time and effort as the system is slow to change. Interviewee 7 shared their experience:

I think there’s a big gap in terms of accessing mainstream services, because the homeless sector is very responsive. And there are some really, really good people and teams doing really, really good work [but ] I think that there could be more coordination across the system. And I think with the resources and the secure funding, we can get there. – Interviewee 7.

In a new context then, where NGOs primarily take on the responsibility of filling service gaps and fundraise to meet these needs, then their independent services will identify and prioritise the needs that they observe. And the final outcome is that when services are provided in a decentralised manner by fragmented services without feedback loops, there is a lack of a system-wide understanding of the current needs being met on the ground. As such the system at a national level does not adequately plan for or meet the needs that exist. Something of a vicious cycle can arise from the lack of mainstream prioritisation of specialist needs where specialist services then step in to fill gaps but then inadvertently mask parts of the full picture of the need that exists. Interviewee 7 explained:

. some of the. . bigger homeless agencies, they have really good fundraising. So there’s naturally going to be additional resources in the system. And we don’t have any understanding of where that, you know, where what kind of services will be set up. It’s entirely independent. . of the public health system. – Interviewee 7.

### CMOC 4: health system values

**Fig. 6:**
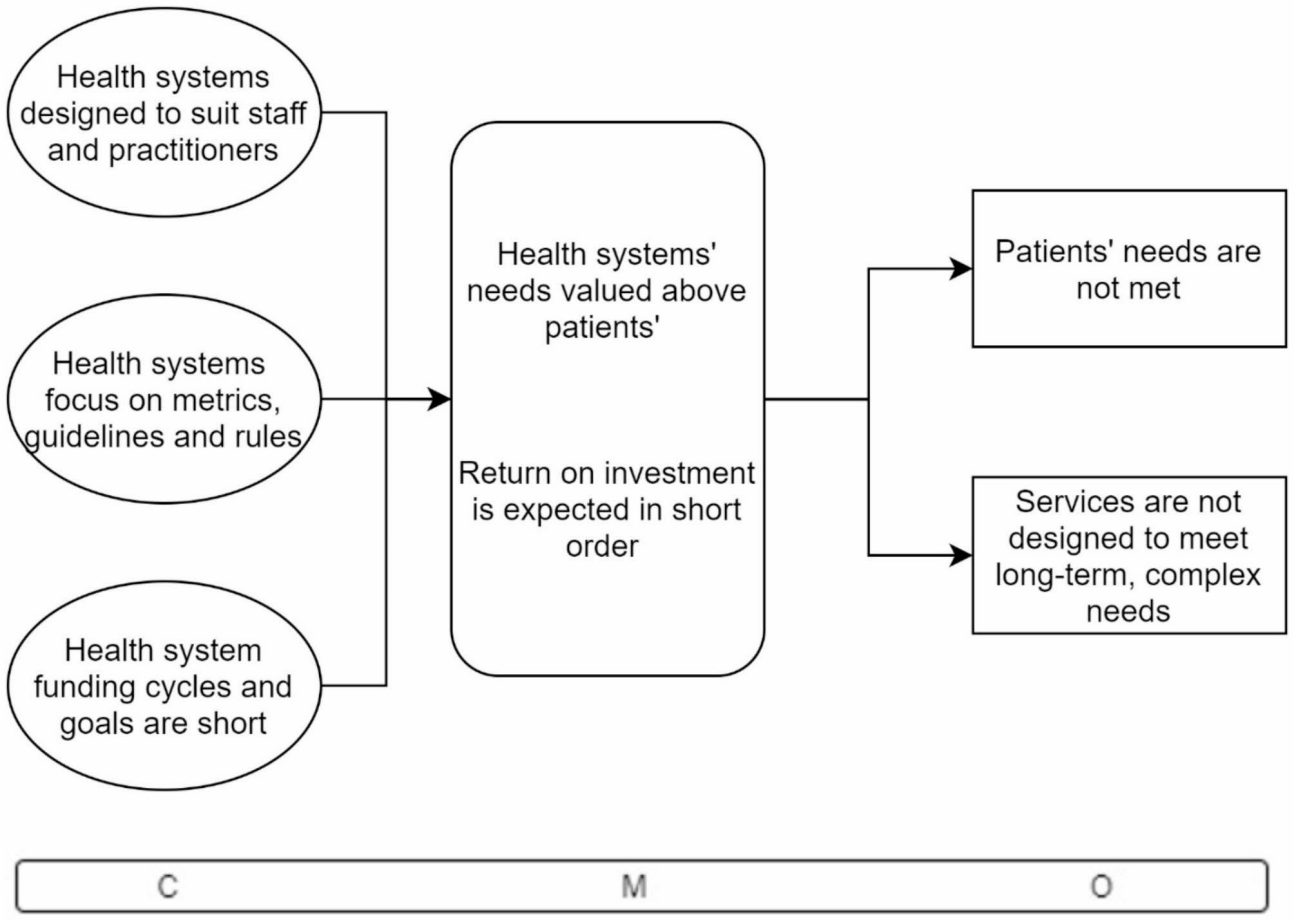
CMOC4 Health system values

CMOC 4 (Fig. 6) brings together three contexts which trigger the same mechanisms. The first context shows that health systems are generally designed to suit the needs of practitioners and staff with regards to their service locations, appointments times, clinic set ups that are suited to clinical work rather than patient comfort, etc. A second context is the focus that health systems put on particular metrics like bed days, length of stay, costs, etc. and the guidelines and rules that everyone must follow in healthcare settings. The third context is where health system funding cycles are short and goals are to be implemented over a short time horizon to demonstrate their success, in the hope of gaining continued funding. These contexts are explored in the following quote:

. particularly in socially excluded people, and it goes right through so in terms of service delivery, how the services are delivered, to what time the clinic is at where it is, how you accesses it, how you make appointments, health workforce, in terms of training, in terms of who, from which social groups doctors come. . frequently you’d see that maybe the catering staff or the porters who would be from a poorer background [and] the socially excluded patients are much more comfortable with them and will already feel that there’s a power imbalance when they’re dealing with the people that are in the health workforce [from higher/different social classes]. – Interviewee 1.

The mechanisms that arise in these contexts are twofold: First, health systems’ needs are valued above those of the patients – the ability for clinicians to practice how they see fit in the interest of making their patient well comes before the comfort and security of patients and the other needs in a patient’s life that may be of greater concern to them in the given moment. Interviewee 5 spoke about this conflict:

If you’re really focusing on what, what do you think is important. . and your needs, and [that] ‘I need to be able to discharge the person without any problems’ and ‘I don’t want to get into a report and don’t have to call the guards’. And I don’t want to have to do all of those things. And if [the patient leaves] I have to do all of those things. And that’s. . your focus, which is crazy. So, I don’t know - person centred care is very abstract, isn’t it?. . I think identifying [patient] needs is massive. And it’s not what we think their needs are. I think we’re really good at that as nurses and doctors. We’re really good at saying this is what you need but actually maybe it’s not. It goes against your instincts [as a clinician]. – Interviewee 5.

Second, return on investment is expected in short order and complex cross-discipline health and social care investments do not fit. Interviewee 1 spoke about the consequences of the short-term nature of funding for services:

And then financing is a huge part of it. . the way that budgets are set up so that health is in one place and social care is in another, the way that … decisions are made on financing based on very short term recuperation, whereas. . you might invest now in early childhood interventions. . and you might see the benefit of that in terms of health 40 years down the line, and we don’t capture that. – Interviewee 1.

Interviewee 8 also spoke about viewing patients through the lens of their life experiences and the critical importance of understanding the effects of trauma:

[W]hat I see in terms of in the healthcare side of things is because for me, everything is very much embedded on top of each other. I can’t talk about healthcare without thinking about early childhood care and what was received for the person because no matter whether you’re homeless or housed, your early childhood care and attachment with your caregivers will predicate how you’re able to look after yourself. For me, it’s more so about people that aren’t able to engage in a system that doesn’t take into account where they’re coming from, where they’re at. – Interviewee 8.

The outcomes are that patients’ needs are not met because systems and services are not designed with their complex, long-term needs in mind. This is particularly important for populations experiencing homelessness or other forms of social exclusion but it is also important for other populations who more often can navigate mainstream health systems with success, because all humans have complex needs and have health outcomes that result from myriad factors that develop over the life course.

### Overarching programme theory

**Fig. 7:**
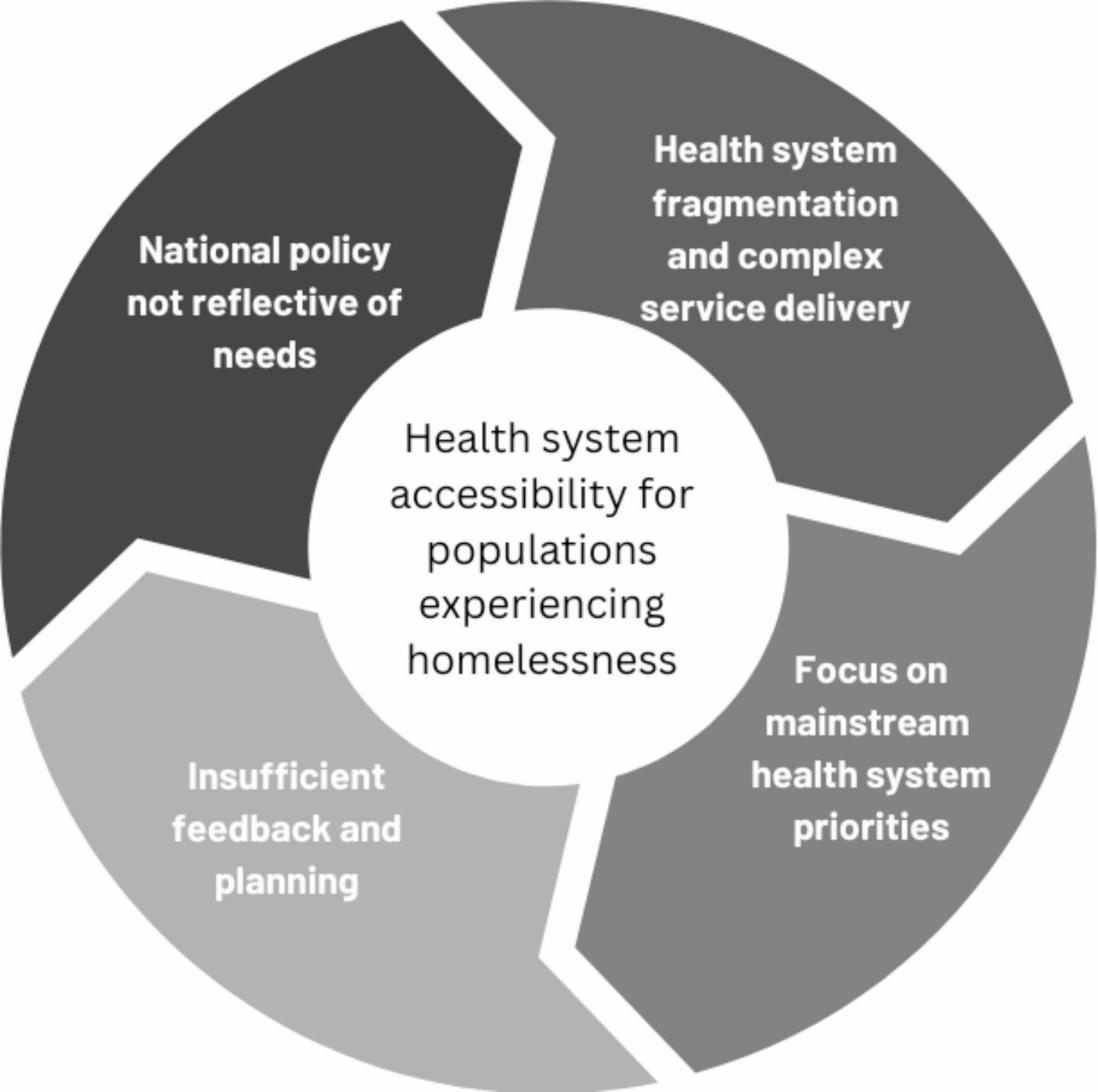
Realist Evaluation overarching programme theory

Synthesising the four CMOCs presented in the sections preceding this one, we have constructed an overarching programme theory (Fig. 7) explaining the full findings of our study. It shows that when health systems are overly complex and fragmented, both between and within services, they focus on prioritising their own needs and as a result specialist inclusion health needs have to be provided for elsewhere. As a result of the default outsourcing of some needs, the system as a whole misses out on information as feedback loops do not provide the full picture of activities and needs for system planning. Incomplete information is fed into high-level policy decisions about whether or not to officially prioritise the needs of homeless and other socially excluded populations. If these needs are deprioritised, it supports the maintenance of a fragmented healthcare system which relies on their needs being met elsewhere.

## Discussion

Building on a realist review by the same authors [42], this realist evaluation sets out to further and more fully investigate two of the six CMOCs resulting from the review. The aim of the study was to understand how funding procedures and health system performance management impact service settings, the staff and practitioners that operate in them, and their ability to make services accessible to populations experiencing homelessness and complex needs.

As described in our overarching programme theory, this realist evaluation found a cyclical relationship springing from the degree of fragmentation in a health system. When health systems are fragmented without tools and means of collaboration and integration both between and within services, health service delivery becomes overly and unnecessarily complex. In a complex, fragmented setting, health systems prioritise meeting their own objectives such as limiting costs, focusing on bio-medical needs over social and mental health needs, and asking patients to fit in with the schedules, locations, and culture of the health system rather than understanding and addressing full patient needs. As a result, patients with complex needs must be provided for elsewhere, or they go without care altogether. Their care is outsourced to responsive specialist practitioners and organisations leaving the system as a whole unaware of their particular needs and missing pertinent information needed for future, more adequate, service planning. Incomplete information is also fed into high-level policy decisions potentially leading to the de-prioritisation of the needs of socially excluded populations. As these needs are deprioritised, health systems continue to cater for organisational needs resulting in the maintenance of a fragmented healthcare system, and the cycle continues.

The findings echo those found in other similar studies. Goodman and Gatward [46] found that deprived populations are underrepresented in epidemiological surveys potentially introducing bias and making them less valid. Similarly our study shows what happens when health system planning is not based on adequate data to fully understand healthcare needs in socially excluded populations. Goodman and Gatward’s study shows that this phenomenon exists internationally and that bias is introduced into the health system at such foundational levels as understanding basic information about population need [46].

Cornes et al. published a realist evaluation of care transitions for people experiencing homelessness being discharged from hospital. Their findings provide an example of how to move forward to bring together parts of fragmented systems to arrange care around the individual. They suggest that safe and timely care depends on ‘localities developing complex adaptive systems’ with clear protocols for discharge planning and patient flow, using clinical patient in-reach, building on discharge co-ordination by multidisciplinary teams with both housing and clinical support, and the availability of step-down care in the intermediate term [47]. Their study offers lessons for service designers to meet the specific additional needs of populations experiencing homelessness to access equitable care within a universal, integrated health service where they may still need expert care and special attention due to their specialist needs. Our realist review [32] highlights the range of additional needs for populations experiencing homelessness and how to meet them.

Prashanth et al. published a realist evaluation of a capacity building intervention in two sub-units within a district health system in India. The study found that health systems strengthening interventions, such as capacity building, works through ‘aligning or countering existing relationships between internal (individual and organisational) and external (policy and socio-political environment) attributes of the organisation’ [48] and that programme designers should identify opportunities for alignment at the design and implementation stages. These lessons would likely be key were a national strategy to make healthcare accessible for populations experiencing homelessness and social exclusion to be implemented in setting where health systems are inward-focused and fragmented and not prone to systematising solutions developed at the frontline.

### Strengths and limitations

A strength of this study is the in-depth analysis of findings from the realist review published by the same authors, utilising CMOCs from that study as the initial programme theory to further uncover causal patterns within a subset of the review findings. Furthermore, the data collected through the interviews represent a range of perspectives from individuals with high levels of expertise in working with and planning the care for populations experiencing homelessness in healthcare settings.

A sufficient level of data saturation was reached to inform the development of CMOCs. The perspectives of the interviewees represent those working in Dublin specifically and the data therefore represent a particularly Irish experience. However, this is common for realist research where causal patterns based on specific data are brought to higher levels of abstraction which apply more widely than the particular setting in which they were gathered. The findings in this study likely apply to similar settings in high income countries where healthcare is predominantly provided by the state.

Additionally, another strength is that while populations experiencing homelessness are the focus of this study, these can be seen as a stand in for other socially excluded populations and the findings in this study also illuminate challenges which populations groups such as drug users, sex workers, migrants, and others, experience in accessing healthcare. Some of these are also experienced by mainstream populations when seeking to access healthcare in a health system that is fragmented.

Limitations include the lack of data collected from service users with a lived experience of homelessness which was not possible because of the pandemic. We sought to counter this limitation and to bring a service user perspective into the analysis by conducting two individual video-conference consultation with people who have personal experiences of homelessness and who currently work in homeless services.

The COVID-19 pandemic presented a challenge to the study in several other ways. First, it meant that interviews had to be moved online which might have changed the dynamic between interviewer and interviewee. During the remote interviews, with some interviewees working from home, there were interruptions such as dogs barking, children needing attention, and deliveries being made. Clinical staff were interrupted by co-workers who did not realise they were in the middle of an interview because they were in their usual workspace seemingly working on their computer. The pandemic also meant that all interviewees were especially busy. However, in all cases they graciously made time to share their expertise.

Another challenge of the pandemic has to do with the data collected. It is difficult to know to what extent the experience of working through such a disruptive and unexpected set of circumstances meant for the perspectives and opinions offered by the interviewees. While the interview questions did not specifically ask about the pandemic, some answers were coloured by the experiences of providing care during it.

## Conclusions

This study has described the methods, analysis, and findings of the realist evaluation resulting in four CMOCs and one overarching programme theory. These findings provide an explanation of how fragmentation and health systems’ funding arrangements and goal setting impact healthcare accessibility for populations experiencing homelessness, based on data collected from 13 interviews with 12 people (as one participant was interviewed twice).

Findings show that there is a cyclical relationship where health system factors that have a negative impact on healthcare access for populations experiencing homelessness reinforce each other in a vicious cycle. Fragmentation leads to complexity in service delivery and in response a health system focuses on its own needs to the detriment of patients. Specialist services and specialised practitioners must fill the gaps and lead the provision of care from the ground up. Due to the fragmentation and reliance on NGOs to fulfil central health system functions, key information is not fed back to the health system as a whole for planning and resourcing purposes. As a result priorities are made without adequate information and the needs of homeless and other socially excluded populations do not rise to the top of the agenda.

To reverse this vicious cycle, health systems can address fragmentation through integration of services with the patient at the centre, focusing on the patient’s needs and their experience through the system. Funding and incentives which promote patient centred outcomes should be in place rather than ones which focus on the system’s need to reduce costs and arrange service provision to suit the needs of staff.

### Breaking the cycle

While most high-income countries have policies and services directed at populations experiencing homelessness and other forms of social exclusion, a focus on the total policy environment must accompany those more focused policy initiatives in order to reorientate the culture and practice of service provision. As this study has demonstrated, it is not enough to have individual services or practitioners meet the needs of socially excluded populations as that can contribute to overall service fragmentation. Rather the system as a whole must be in position to understand the full needs of all populations groups in order to employ resources where needed. Within the health policy domain specifically, health services should be universal, integrated, and organised around the needs of the individual person. Additionally, the role of stigma and trauma in the experience of healthcare access for populations experiencing homelessness must be widely taught throughout the health services.

Government and high-level health system leadership should take the following measures to enable healthcare services to become more accessible for populations experiencing homelessness:

- Develop a national strategy to guide integrated, coordinated health services to make the health system responsible for recognising and responding to social determinants of health with adequate funding and specific goals attached.
- Provide all practitioners and staff with adequate training and exposure to socially excluded populations to develop a baseline of knowledge and expertise in providing appropriate services to them, and provide expert training to specialist practitioners.
- Develop and support trauma awareness and understand the role of trauma in homelessness, social exclusion, and health.
- Embrace definitions of health that are not narrowly focused on bio-medical outcomes but holistically embraces all aspects of health depending on patient wishes at the time of a clinical encounter.
- Adopt a flexible and inclusive culture championed by leadership at every level of the system.
- Provide multi-year funding for health services.
- Enable practitioners to organise services around the needs of patients by creating service structures and pathways with patient input, and through empowering practitioners to respond flexibly to the situations in which they provide care and need to act outside of the typical set of procedures.
- Take responsibility for meeting the needs of all populations and put into place tools for gathering pertinent information for continual planning to adequately meeting changing needs on an ongoing basis.

These recommendations will benefit all people using health services and as such investing in them would pay dividends for populations beyond those experiencing homelessness.

### Electronic supplementary material

Below is the link to the electronic supplementary material.

Supplementary Material 1

## Data Availability

Data used to draw the conclusions presented in this article can be found in the supplemental file.
