# Supplementary material for: How do health system factors (funding and performance) impact on access to healthcare for populations experiencing homelessness: a realist evaluation
Source: Int J Equity Health. 2023 Oct 17;22:218. doi: 10.1186/s12939-023-02029-8 (PMC10583475; doi:10.1186/s12939-023-02029-8)
Supplement: Supplementary file 1 — Supplementary Material 1 [file 12939_2023_2029_MOESM1_ESM.docx]

RS et al (2023)
How do health system factors (funding and performance) impact on access to healthcare for populations experiencing homelessness: A realist evaluation
Supplemental file

**Consolidated CMOC 1**


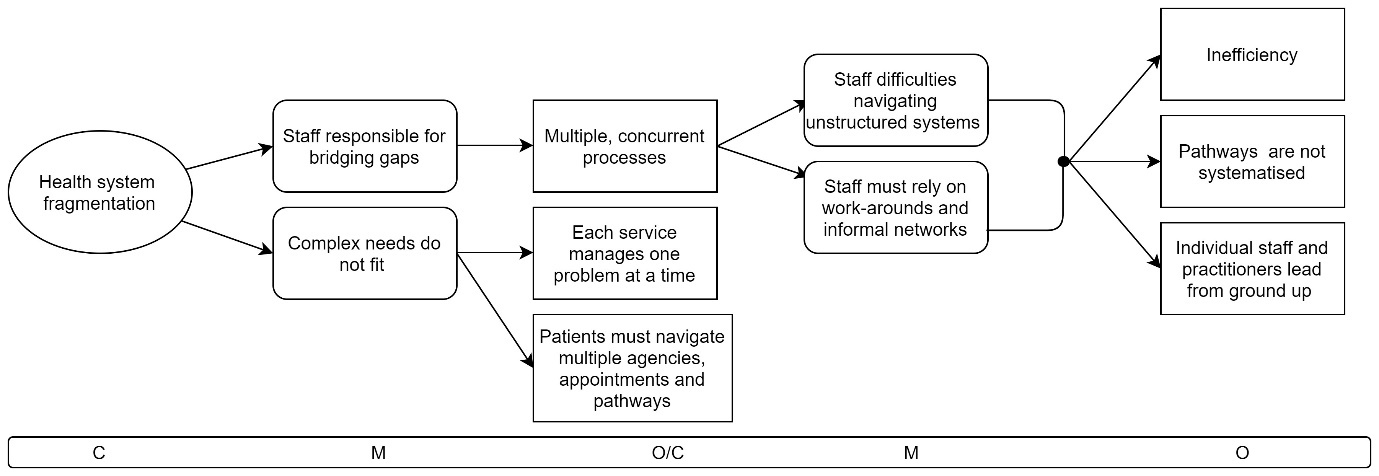


Supporting data:

“And usually, I think that's not intentional, but it's unintentional, but just that their complexity and the way that we think I suppose the biomedical model where it's one disease that you're treating doesn't fit in very well with social exclusion, where people have multiple diseases, multiple things going on, I would actually argue that that one disease at a time fits very few cases that very few people, but particularly in socially excluded people, and it goes right through so in terms of service delivery, how the services are delivered, to what time the clinic is at where it is, how you accesses it how you make appointments, health workforce, in terms of training in terms of who, from which social groups doctors” – Interviewee 1

"And I think the more we set up this, this idea that, and support and resource, a safety net, rather than primary care and non-fragmented general practice, I think the more fragmented healthcare, you get. And I would be of an opinion that we need to do more to fix the fundamental issues and gaps, as we've been articulating, with the first port of call, rather than buttressing up a safety net, that can just fragment care further, and cause more division. And I think for a variety of reasons therefore, it is imperative to support primary care and general practice first, to absolutely call it stigma amongst healthcare professionals and say it's uprofessional and have sanctions. And then, after and in parallel with that obviously support a safety net. And then in the safety net to actively support someone getting back into general practice, which I don't believe is really happening. I think there are malincentives". – Interviewee 3

“It has cost me a lot of fall out professionally with other clinicians trying to access care for my population because mainstream services, follow the guidelines and they follow strict appointment times and well you didn't show up three times so now you have to go back to the doctor and I'm like, grand, I'm writing the referral right now, no you give them another appointment like you're just making me go through a process that doesn't need to happen because somebody said that you needed to do it. But why are you doing it? . . . Does it affect the behaviour of the person, does it actually increase the availability of the service, or your waitlist to be addressed? Like, it's just an arbitrary rule. And I think that more mainstream services that do that continue to exclude our population and what we try to do, is we adapt the guidelines. You know, we consider the guidelines and use them to the best of our ability to meet the needs of a population that the guidelines aren't written for.” – Interviewee 4

"It is so much easier when I have one contact in this department, I have one contact here and you have the people that you contact and they know where you're coming from and they know where you're coming from". – Interviewee 4

"I think specifically the hospital and this has always been something that I've done, because I've worked with people, for a long time that don't go to hospital don't go and like traditionally it's the epilepsy clinics, the hepatology clinics, the HIV clinics. So, for about 10-15 years I have colleagues in those sites. Now so I have the networks, I have the experience". – Interviewee 4

“As much as it like has seen huge amazing, amazing progress in terms of awareness and things like that but, it is very much based on individuals, and those individuals when they're not there, you know. . . And I suppose it shouldn't be like that because that's how it fails, you know. Because it's. . .maybe I'm being too harsh, but I feel like if it's based on me or [name of nurse] or [name of nurse] or [name of doctor], I think you know I think there has to be driving forces in leadership. They should be able to function without us and I worry that wasn't the case. Because the healthcare hasn't really taken it on as part of their system. It relies a lot on going okay, would you fix that grand, push it over there. . . I think they're happy to have people that are doing these system pieces. You know they can get outcomes, even better, and if we don't have to finance much, even better. And we can show improved health, great, but if those people aren't there you'd wonder, are the systems there to keep it going?” – Interviewee 5

"So okay, so systems like that. I find the MDT [clinician/staff-led multi-disciplinary meeting with staff and clinicians from across Dublin health, housing and social care services], I find what it is, is that you have a lot of like-minded, various experiences around a table. And I find it works and I've kind of adapted that, we've adapted that to other areas. So like what I was saying like the diabetic foot service, like we have a little MDT every week. And it's not that we're going to change the world with it but all our eyes on this particular person for that, for that be it five to 10 people in various circumstances, opportunistically. So eventually when that person lands in the ED, there's a note on the system, it's like you know, get the podiatry service over to them you know, as when you can because they've been trying for it and then if they can, at that point, they'll come over". – Interviewee 6

"And I can see when it works, I can see where if you had more time, you could do more work, erm but very much the system, you know, you've said, it's been said so many times, but now I'm starting to quote it, you know, erm you break your leg or whatever, and you come into hospital, that you have somebody who's going to drop in your stuff to you, you've someone who's going to pick up, you're going to have a phone, you're going to have contact, even in COVID times . . .you leave the hospital on the day, someone's going to pick you up or will organise it and you have a pharmacy to go to you, have money to collect them. And it's just all those simple things. . . the cohort, particularly I suppose the emergency accommodation people that we would be looking after or even those, it's very overused, the word chaotic, I suppose. But people who have a lot of things going on in their lives. They even more so have more things to be thinking about while they're in here and more worries. And they just don't deal with that system. The system just is not made out for them. So just off the bat, which I know you've heard, but working here you see it.’ - Interviewee 6

". . the concern is if you have someone who, with a disability, for example, who's homeless, or they've always had a disability, and then they become homeless, and then the next minute, they're serviced by the homeless, erm homeless health teams, which doesn't make sense, because just because they lose their home, it doesn't mean they can't still access disability services. It's that kind of so it's kind of like, you know, the mental health addiction thing. You can't fit into one box, I think every service is overlapping and comorbid conditions, I think every service has to be aware and considered of those and also of the kind of social determinants". – Interviewee 7

“So for me, everything in terms of systems come from the top down. Because we're an acute system we're not a grassroots organization that are starting something and changing something. Whereas Inclusion Health are trying to be a grassroots organization within a hierarchical structure. So for me, it's all around the culture of the organisation, and how that sets up things for the whole population group. So, for example, the leadership within the hospital system would not necessarily be well versed in the additional needs, some patient groups have . . .” – Interviewee 8

‘If I'm working with a client [in mainstream health services] they'll know me, they'll build up a relationship with me - I'm [name] I'm their [practitioner]. . . That doesn't happen with homeless people that you work with because they have so many people involved . . . like oftentimes they will, but there'd be a portion of them that will get confused - who are you again?” – Interviewee 9

“I think one of the stand out aspects of this sector is the interagency work. So that collaboration is different to what you see in other mainstream health services. And I think purely because it has to be. Erm I think the need kind of leads us a little bit but it's a good approach to have" – Interviewee 9

“Yeah, I think that the way we prioritise our service delivery model is based on what's good for, what works for the 70% of society, who go to work, and can keep appointments. Erm obviously, the people who design things come from that cohort. . . appointment times, 10 minute consultations, type of clinic, the way even a clinic looks is built for the 70% of society. It's not built for the drop-outs of society. So obviously, it doesn't meet their needs. Erm they can't keep appointments, don't prioritise healthcare. Erm the system we have built, they weren't involved in . . . They weren't involved in commenting on the design. So obviously, it doesn't work. It works for the 70%. Erm, and excludes the others. So that's the problem. That's a barrier in the service delivery” – Interviewee

“Erm I found that by following [in research work], you know, the primary care practitioners, public health nurse, GP, the different services, by hanging around I actually saw the same person, that's how I know, when I say to the person who looks like seven people to the system I saw the same person coming in, in this domain. And then I was in another domain, the actual same person, and the two services had never spoken, and the three services and then in another domain, so that the problem of that homeless person or the drug user looked far more complex from the fragmented service because it was fragmented.” – Interviewee 12

“You know, homelessness is being looked at from an accommodation point of view which is all ridiculous. And, and accommodation isn't married with health. [Inaudible] there is a whole problem with the system at the moment is erm it's reactive . . . and if something goes wrong and a workaround is created and work arounds are talked about as if they're positive things. And the whole thing is now clogged up, because so many workarounds you can't even see what the system, the thing's supposed to be in the first place. And it's weighed down with the workarounds and then work arounds on top of the work arounds.” – Interviewee 12

**Consolidated CMOC2**


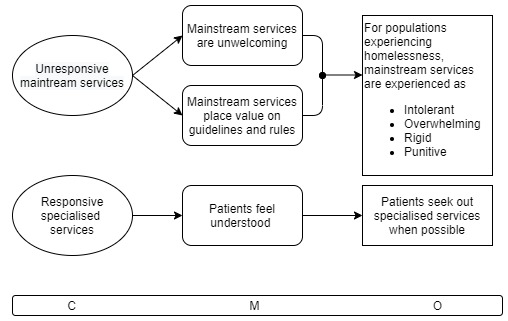


Supporting data:

“So I think so there is a huge piece around the philosophy or the psychology of the health system and how it decides who's worthy of care and the way that it responds to addiction or certain behaviours. . . And I think that probably is a systems thing, because it's built into the system and it's reflected through the system, but then also a lack of flexibility. So I think sometimes people who design healthcare see it as like a factory, a conveyor belt, erm, but that model doesn't fit to people who have a lot of other issues going on. And so that would be another big barrier. But to be honest, I think actually probably the most important pieces around who deserves what and how, how our perception on some level that feeds very much in at a system's level that if you punish people, or if you help people that or make it easier for them to access things that you're rewarding bad behaviour, and they're going to engage in more of it. And I think that's partially why the system is very punitive”. – Interviewee 1

“I do think there are certain skills in managing, you've got, this is a highly traumatized population. And so a lot of their issues are behavioural issues. . . so you build up a set of skills for working with those people. But . . . you have to be really careful that people get caught up with your service. So in other words, if someone is attending a homeless service, and then they go back, they get accommodation in the community. Over the next year, you should wean them off your service”. – Interviewee 2

“It has cost me a lot of fall out professionally with other clinicians trying to access care for my population because mainstream services follow the guidelines and they follow strict appointment times and well you didn't show up three times so now you have to go back to the doctor and I'm like, grand, I'm writing the referral right now, no you give them another appointment like you're just making me go through a process that doesn't need to happen because somebody said that you needed to do it. But why are you doing it? What's it based on how? Does it affect the behaviour of the person, does it actually increase the availability of the service, or your waitlist to be addressed. Like, it's just an arbitrary rule. And I think that more mainstream services that do that continue to exclude our population and what we try to do, is we adapt the guidelines. You know, we consider the guidelines and use them to the best of our ability to meet the needs of a population that the guidelines aren't written for”. – Interviewee 4

"And I think that’s where, cause there’s some practitioners in homeless health that aren’t accessible. There’s some that don’t demonstrate the values that or the same commitment or the same approach. It’s definitely on a spectrum even within our services. There’s some people that have terrible times with nurses and doctors and there’s some that they would prefer to go to because they know they’re met with a certain approach. But what I’ve learned is that… It took me a long time to really realise is that it’s not my agenda. It can never be what I think they need to do and as soon as you step back from, you know, well, if you stop drinking then you won't have this bleed and you won't end up in hospital, and therefore you won't die . . . as soon as you step back from erm, you know, thinking that they need to, it's task oriented for you as a clinician, and that somebody needs to stop doing this so that they become healthy. Then, I felt that it relieves all that pressure and all that anxiety on me to make that person change. . . My job is to just support them to understand the impact of those things. And to provide them with alternatives should they want them, but also provide them with alternatives to keep them as healthy as possible while they still do the thing that's detrimental to their health. So, regardless of the fact that you're still drinking alcohol, and that it's really bad for you, we're also going to look after you know your nutrition, we're also going to look after, you know, we're going to give you your flu vaccine and we're going to do all these things for you. The focus isn't always going to be on the one thing that is, is the bad thing that you're doing . . . Healthcare providers need to be flexible, opportunistic. It takes like 5000 more tries to do one thing. And it's those little little wins that are the success stories. People always ask me isn't it sad that people never stop or that they never get better, and you know it's little things like oh my gosh they actually went to that appointment today. That took me nine, that's nine months of not nagging them but gently, you know, you could have six failed attempts to get somebody to this one place and then they finally go, and it's just about the fact that you need to be creative, that the usual pathway that you follow or how things are supposed to happen. You need to understand that that's not going to happen that way. You need to be really creative in how you bring that support to the person or how you get that person to the support and it takes a long time". – Interviewee 4

“I could pick out a few individuals that still come in and the staff will still say oh, they're asking for you and it's not about me, like I know that it's not about me but they, they know that I know their whole story, you know, they don't have to explain themselves. You know, I think you know the needs and there a lot of sort of yeah that trust is really important. And I think, it's being really adaptive. I think in health care, in the hospitals anyway, we're very focused on rules, and it doesn't work for this group . . . like if you're saying ‘you have to sit there until you're called’, like, it's like a red rag to a bull. If you've got somebody if in an ideal situation, if you had somebody to deal with individuals or even two or three individuals, it would definitely would help and it takes away from the triage nurses or the nurses on the floor taking huge amounts of stress and workload on them because just sorting out their methadone, that's what they feel is their biggest need, and if you can sort that out then you might get another hour out of them”. – Interviewee 5

"From my perspective, I think there's a big gap in terms of accessing mainstream services, because the homeless sector is very responsive. And there are some really, really good people and really good teams doing really good work. Yes, I think that there could be more coordination across the system. And I think with the resources and the secure funding, we can get there. I do I feel optimistic that we're on the right track. I think that concern, from my perspective is that often the, if there's an issue, and it's related in any way to homelessness, it's lumped on Social Inclusion. So what you have is then the other parts of the HSE, not responding to certain minority groups. And this is not just homelessness, it's across all social inclusion groups. And I think that's where there's a real gap, because there has to be more sort of policies and mandatory kind of training and mandatory responses to ensure inclusivity to ensure that mainstream services are inclusive, and that just because they're homeless, they shouldn't be sent to a homeless team, because that's not necessarily needed. And it can create exclusion”. - Interviewee 7

". . the concern is if you have someone who, with a disability, for example, who's homeless, or they've always had a disability, and then they become homeless, and then the next minute, they're serviced by the homeless health teams, which doesn't make sense, because just because they lose their home, it doesn't mean they can't still access disability services. It's that kind of so it's kind of like, you know, the mental health /addiction thing. You can't fit into one box, I think every service is overlapping and comorbid conditions, I think every service has to be aware and considered of those and also of the kind of social determinants. – Interviewee 7

". . you don't want someone being trapped in a specialised service and being kind of excluded, in a way, I suppose. And not being able to go back and access mainstream services". – Interviewee 7

“So, erm the other, one other thing I suppose that we find is a massive barrier, and when it works it works so well, is interagency working. So in homelessness [services] by its nature, you usually have a lot of services involved. So whether it's your outreach workers on the streets, the people who serve the food in the soup kitchens, it could be the drug treatment workers, then you've got the mental health workers which are us, the accommodation providers. So there often be about maybe 10 different organisations involved. If those organizations communicate with each other, it can work really, really well. When they don't communicate with each other, it can be so difficult, because you've got different things like people doing the same kind of work from two different angles, particularly if you've got somebody with a mental health condition where they maybe have traits of personality disorder or something like that . . . where they're not fully erm capable of managing their own affairs. They could have two or three different organisations working towards the same piece of work with them. And if those two or three organisations aren't communicating with each other, it can become really chaotic.” – Interviewee 9

“. . it shouldn't be a separate homeless service until it comes very far down the line. And you have to be very careful... But if you are going for funding for erm your specialised services, which is for homeless as a separate thing . . . obviously it's not going to work perfectly and you're going to need additional funding. But you should never be setting up erm parallel systems or structures for homeless people. Erm it should always be erm with the intention that erm, you know, or the acknowledgement that homeless people are not separate from the community because otherwise you'll end up with an apartheid system and another apartheid system - two-tier: public, private and then homeless.” – Interviewee 12

**Consolidated CMOC 3**


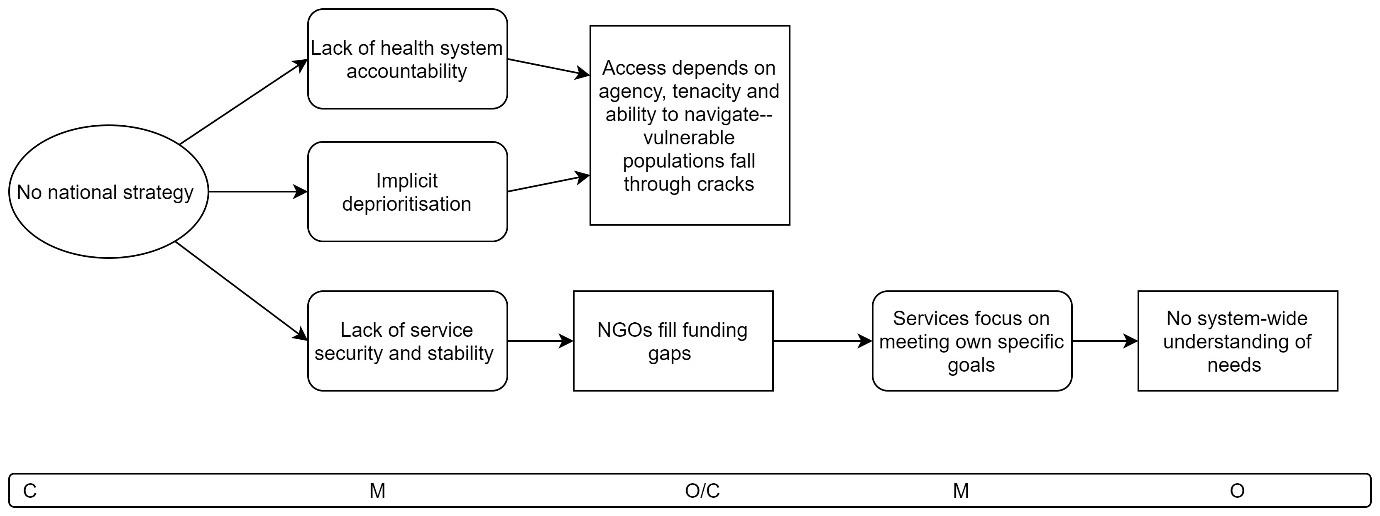


Supporting data:

"[Medical cards], they are whilst it is notionally free and accessible erm the long waiting lists to access psychological services, counselling... I got someone who was recently shot nine months ago, recently, bereaved when his brother died, addiction, multiple traumas he needs to get counselling and its never available ever for that person. So, so I think all other persons who have medical cards, may have the resource and the sticking power in terms of, you know, the psychological capability to be able to stick with it. But these persons can't advocate and I think that the absence of those services renders wider primary care services notionally present but practically absent". – Interviewee 3

“Healthcare providers need to be flexible, opportunistic. It takes like 5000 more tries to do one thing. And it's those little little wins that are the success stories. People always ask me isn't it sad that people never stop or that they never get better, and you know it's little things like oh my gosh they actually went to that appointment today. That took me nine, that's nine months of not nagging them but gently, you know, you could have six failed attempts to get somebody to this one place and then they finally go, and it's just about the fact that you need to be creative, that the usual pathway that you follow or how things are supposed to happen. You need to understand that that's not going to happen that way. You need to be really creative in how you bring that support to the person or how you get that person to the support and it takes a long time". – Interviewee 4

"I think on a national level or even an international level when you look at all the documents and the policies thoroughly, they all talk about homeless populations as a vulnerable group, and the emergency medicine program in 2012, I think it was, they identify homeless people as a vulnerable cohort. And yet, like, what, what do we do about it then? Like if you call something vulnerable and say that their needs need to be met. I think you have to ask them those things”. – Interviewee 5

"And that's where I think, certainly, if there was an inclusion health strategy, or a social inclusion strategy, or whatever you want to call it some strategy, or public health strategy, where there is also that kind of expectation that it's the responsibility of the entire system, to recognise, be aware of the social determinants of health, be aware of certain risk factors or vulnerabilities that people would have, and how to ensure that systems and their services are set up to respond to that, and to ensure that everyone feels included. I mean, beyond homelessness, there's issues like interpretation, having access to interpreters in some services, and yeah, there's a lot of things that you know, services aren't set up always to support these minorities and they should". – Interviewee 7

"It would be accurate for that time that I've been there that the funding stream, a lot of the funding will be once off. It's not attached to any sort of long-term strategy". -- Interviewee 7

"Yeah, NGOs? I mean, the HSE will, the majority of our funding will go to like section 39 [organisations], which is voluntary agencies . . . so to some extent, you have some oversight about how the system is coordinated, so to speak. But having said that, you know, some of these organisations now, like, [name of NGO] . . . the bigger homeless agencies, they have really good fundraising. So there's naturally going to be additional resources in the system. And [the mainstream system] doesn’t have any understanding of where that, you know, where what kind of services will be set up. It's entirely independent, I suppose of the public health system". – Interviewee 7

"No. There's no big overall thing. There's no targets no goals and no evaluations. There aren't. No. And that's, that's what's needed". – Interviewee 11

**Consolidated CMOC 4**


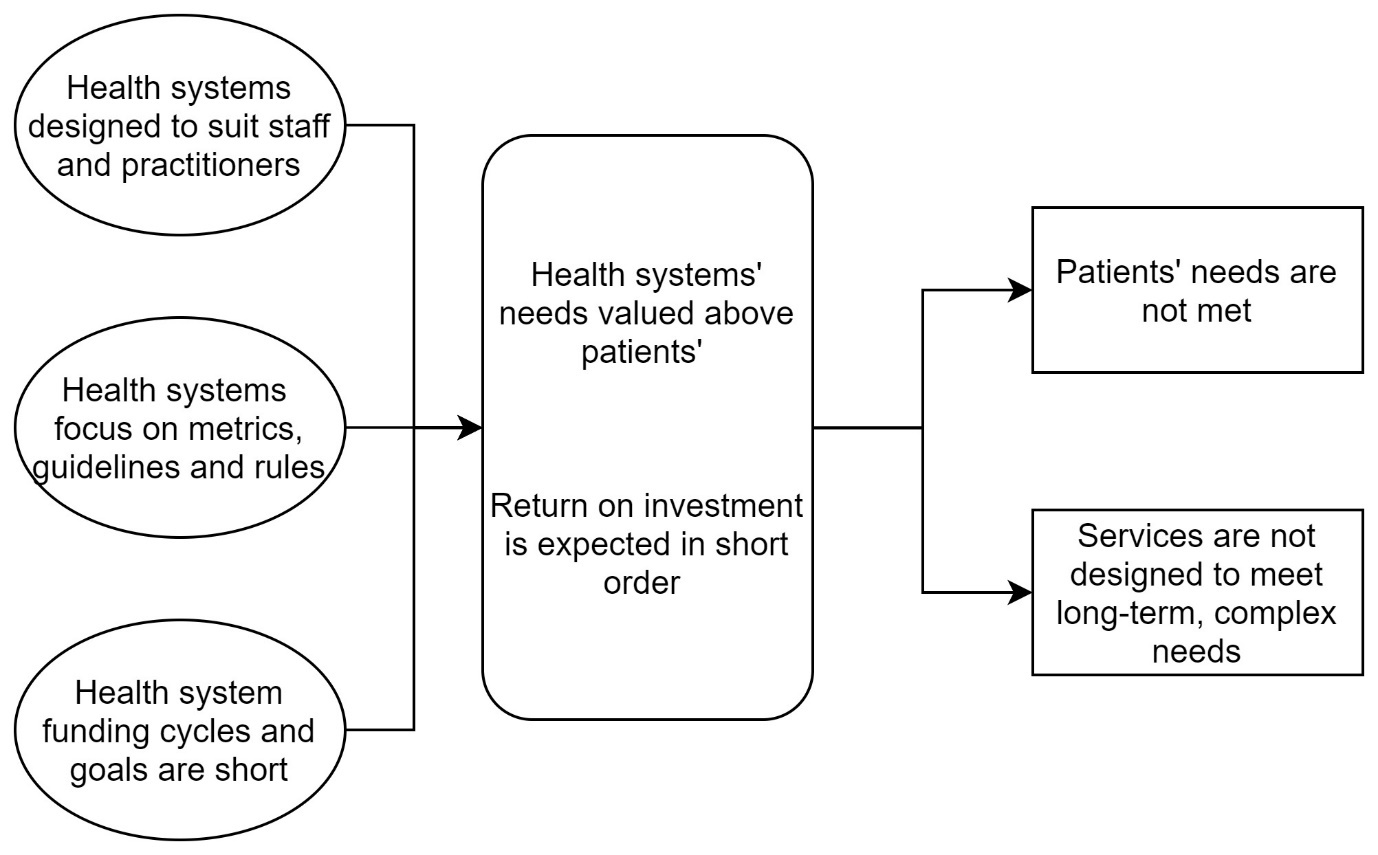


Supporting data:

"And then financing is a huge part of it and we've talked about this before but the way that budgets are set up so that health is in one place and social care is in another, the way that decisions are made on financing based on very short term erm recuperation, whereas, as we've talked about, you might invest now in early childhood interventions erm and you might see the benefit of that in terms of health 40 years down the line, and we don't capture that. And we look at health as a unit of a person rather than the health of the community, which is probably another really important way to be able to look at it. And then that all feeds into there's, it's a system that isn't fit for purpose, I would argue, in treating people, let alone people who are complex". – Interviewee 1

“ . . the mainstream services are not designed for homeless people, they're designed for housed people. And they suit the needs of housed people. They do appointments, they do, you drive in, parking places, you keep regular times, people whose behaviours are not chaotic. And anyone who goes into that system, who feels out of that system, outside that system, will automatically find it difficult. You will find a number of GPs who will make it accessible. But to make the whole service accessible... And in reality, I don't know of any city that has made, able to provide proper healthcare to homeless people through making general practice things. – Interviewee 2

"I think the whole process of applying for a medical card . . . it needs to be framed within the context of what a service user needs and that's quite doctor centric, form heavy . . . So I would often get a call from someone saying 'can you take on these two patients they're both homeless?' And I would say like no problem just tell them to ring my mobile. And they say, no they don't have a home, they don't have a mobile. Well can they call at the practice? No. So, for them to sign up, I have to meet them". – Interviewee 3

“There are things that need to be provided in the hospital, and there's probably 70% of that stuff can be provided in the community, where the person lives with the practitioner that they know, over a period of time. You know, bloodwork doesn't need to be done in the hospital, it can be done here and it can be sent. You know investigations can be done. You know, you don't need to spend eight hours in the hospital waiting for a chest X ray and an ultrasound. You can plan to do them on different days and then collate all the information and then discuss it. So, you know, I think, the next step is the outpatient clinics.” – Interviewee 4

“. . clinical guidelines are exactly what they are, they're guidelines, they're not. They're like they're, they're the best available evidence possible but they don't fit every population, they don't fit every person and they don't fit every situation. And I think as a practitioner, especially as a nurse, you know it's slightly down the hierarchy in terms of the medical field and it's a predominantly female profession. So, I think there's a lot of anxiety there about oh my license, I be a can't be innovative, or I can't flex from the guidelines or I can't do this" - Interviewee 4

"I think that so there's a two prong, there's another approach which is okay, maybe next time or okay, I have to go back to the drawing table and find something more creative or I have to work on my relationship with you over the next seven months before you engage with me on this thing. And I think, you know, especially GPs and some nurses as well. They don't have that time. So it's always an agenda, whether it's a time agenda or an anxiety agenda. Or maybe that person falling through the cracks and becoming very, very unwell and you not having done enough persuade them . . . Accessibility to me, isn't just about putting a clinic in the hostel. It's not just about giving you a taxi to go there. It's about empowering people to understand, giving them the space, giving them the attention so that they can take in the information, and also making sure it's not about your own shit”. – Interviewee 4

"It has cost me a lot of fall out professionally with other clinicians trying to access care for my population because mainstream services, follow the guidelines and they follow strict appointment times and well you didn't show up three times so now you have to go back to the doctor and I'm like, grand, I'm writing the referral right now, no you give them another appointment like you're just making me go through a process that doesn't need to happen because somebody said that you needed to do it. But why are you doing it? What's it based on how? Does it affect the behaviour of the person, does it actually increase the availability of the service, or your waitlist to be addressed. Like, it's just an arbitrary rule. And I think that more mainstream services that do that continue to exclude our population and what we try to do, is we adapt the guidelines. You know, we consider the guidelines and use them to the best of our ability to meet the needs of a population that the guidelines aren't written for". – Interviewee 4

“They [some staff and providers in mainstream health services] also take it as a personal affront. And then it's like, oh, well they didn't come so we're gonna put them to the bottom of the pile. Cause there's other people that need the service and we're very busy”. – Interviewee 4

"I think a lot of these things can also be translated to the care provider - less frustration and fear. Yeah, I think, definitely. I mean I've noticed anxiety amongst healthcare professionals because the person didn't want to see me today and I have the results of a blood test"". – Interviewee 4

“. . there is obviously a limited pool of money and resources and these patients definitely take a huge amount time and that's why I think the specialist roles have just been amazing because I found so willing to engage with me erm when I was in the role because suddenly you are saying, 'I can help you with this'. And you know from being on the floor that, you know what the person might need or what you could do for them, but you just don't have time and I know that sounds really, really dismissive or like you know it's a throwaway comment, but it's really hard when you've got people in resuss or who are really critical or you've got 20 people on screen for triage and you've got somebody shouting at you for their methadone, which is really important we know. And I think that the way they, the way I'm not generalising but the way a lot of patients interact erm because they have really poor relationship with authority and hospitals and they have a really bad experience. And a lot of it's not their fault. I think that comes into every conversation. You know, the minute they come in a lot of people they want things, you know. Erm and I think they feel they're being treated unfairly and inevitably sometimes that happens". – Interviewee 5

"And I think, it's being really adaptive. I think in health care, in the hospitals anyway, we're very focused on rules, and it doesn't work for this group, you know, and if you say you can't do something. So . . . like if you're saying you have to sit there until you're called. Like, it's like a red rag to a bull. If you've got somebody if in an ideal situation, if you had somebody to deal with individuals or even two or three individuals, it would definitely would help and it takes away from the triage nurses or the nurses on the floor taking huge amounts of stress and workload on them because just sorting out their methadone that's what they feel is their biggest need and if you can sort that out then you might get another hour out of them. It may be not why they attended but really they felt their needs are different to what we see as their need. I think we're sometimes very good at telling people what their needs are when actually it's not always the case and you manage to around the back way, you get there a bit easier and you've less arguments and that trust definitely builds so I think that's a really good one. There's definitely less frustrations." – Interviewee 5

"It is and its a lot of like patting ourselves on the back I think, you know and you hear it at the moment, more than ever. And its like come on, like everyone's doing good job. So I think if people feel like that about themselves before, you know, I think before they even go into a person. If you're really focusing on what, what do you think is important, then, and your needs, and I need to be able to discharge the person without any problems and I don't want to get into a report and don't have to call the guards. And I don't want to have to do all of those things. And if you leave I have to do all of those things. And that's like that's your focus which is crazy. So, I don't know - person centered care is very abstract, isn't it?" – Interviewee 5

"I think a lot of time we're taking on the responsibility of this is really important because you have this and this condition and that's not a priority to the patient. But we know it's a priority and services know what's the priority and they're really worried about it. And I think if they've somebody to talk to about it and we know we can talk to them about it I think the right thing, does probably happen for the actual patients but they might be so focused on one part of the puzzle that's not actually improved for them. Erm I think that's a hard one to do because people's priorities are different". – Interviewee 5

"I think identifying their needs is massive. And it's not what we think their needs are. You know, I think we're really good at that as nurses and doctors. We're really good at saying this is what you need but actually maybe it's not. It goes against your instincts". – Interviewee 5

“I mean, [it’s] obvious to me just straight from the bat, that [the way health services are delivered] doesn't work for our clients, unless you have something like an inclusion health [service] and that needs to be bigger". – Interviewee 6

"The issue here is that we haven't had secure kind of recurring funding. It's been once off funding. So it hasn't been mainstreamed, which is it does create uncertainty for organisations and it does create that instability, I think in the system". – Interviewee 7

". . there is a number of different funding streams in homelessness, it's quite complex. We were fortunate enough to get a decent budget this year, through the winter plan and through the National Service Plan. So I suppose everything that we have been pitching for every year, we've finally received, which means that we now have recurring funding. So I mean, like, it'll be interesting to see where that takes the system. And how that will, how that will support the system and improve, potentially, hopefully improve access. But then you, then we still have the same issue where next year, we have to pitch for funding, again, because you have projects like the hospital, not hospital discharge, I think that's mainstream now. But like the housing first project, which is somewhat funded on a recurring basis, but the project will end 2022. And then we need to ensure that that funding is mainstreamed. And programs like that's really important. Because you're, I mean, essentially the project is around time-unlimited support. So if you pull the supports away, or if there's that kind of insecurity that this is supposed to be time-unlimited, I'm supposed to have a house forever. But that no one's really sure of whether that funding is going to just end or not. Of course, that's an issue". – Interviewee 7

"For some of it, I'd say it's the appointment basis of outpatients. So lots of people that have experienced homelessness might be in a little bit of a better setup and might be in a longer term hostel. And . . . they might come ED, and be given a follow up appointment for a different team to maybe follow up with a fracture or just don't know, whatever the healthcare need might be. Those appointments are often a bit, a good bit in advance, a very minimum time slot, so if you don't present at the time you're not getting that, erm, written in illegible handwriting on an outpatient card for people that may have very very poor literacy skills, erm with no directions to where that place is necessarily on it, and also at a very early timeframe. So if you're say homeless and either rough sleeping or in the emergency hostel accommodations where you're out from early in the morning. So you mightn’t have, you mightn’t know where you're waking up that day. Erm you might have to go and try and get your methadone, you might have to go and try and get your payment to ensure you have, if you don't have methadone, we can get another substance that will not force you into withdrawal very quickly. And, and you might have to also avoid lots of people that things have been challenging with around the place. So your day is very busy. People think homeless people are just sitting idle, they're busier than me and you, going about the place trying to ensure their daily needs are met on a daily basis because they've nowhere to put, they've nowhere to store the bag of mementos or whatever it might be. So, I think, a barrier is often those outpatient appointments that might be fine if you were gone into work and saying oh actually I'm going to be an hour late because I have an appointment, and you give your cert and it's on the sick and all of that's fine. Erm, but if you're getting off a bed, getting off the floor at Merchants Quay after sleeping all night on something that is possibly two inches thick like a yoga mat you mightn’t be able to mobilise anywhere, you mightn’t be able to make it to the bus stop, you don't have the funds to get yourself a taxi. You mightn’t have a shower in a couple of days, you might be conscious of how to how you feel, to think I'm not, I don't, I don't look well enough physically to actually walk into a and sitting in a literally be sat on top of each other in an outpatients while waiting to be seen. Erm, like, so there, everything is set up to meet the system's needs to ensure that there's a registrar sitting in the clinic for x amount of hours getting through x amount of patients. That the secretary has put through on the system to give a time slot for 15 minutes each, that's all you get. So if someone is late the doctor has to be there late, and the doctors aren't able to because there aren't enough of them to go around. So I think there's a huge amount of barriers that are not being seen by the medical systems but that are very much very real for people. So for example, if you're homeless and you don't have a watch, do you know what time it is? If you wake up in the morning you and it's bright out you mightn’t know what does it get bright out? Very simple things but very impactful. Or you might find yourself okay well I decided last night it was more appropriate for me to sleep on go sleep on a bus because I didn't want to go to hostel because I was afraid of xy and z person, and I end up in a bus, the bus went out to the depo in Swords and now I'm in Swords and how do I get back? Do I even know what day it is?" - Interviewee 8

“So in the hospital at the moment, what I've seen in terms of the homeless population is that it's very challenging for someone that does not see themselves as worth any care to then be able to engage in a care system that is individual. That is the individual that needs to attend for an appointment once or twice, and then they're off the list. Erm or it's the individual that needs to understand the language that is used by the doctor or the nurse or the caregiver who are looking after them. Because unfortunately, a lot of the people in homelessness have very low levels of education, very low levels of literacy, have high levels of early childhood neglect which results in different areas of the brain not functioning as well as they might need to, to be able to take on that level of information. So what I see in terms of in the healthcare side of things is because for me, everything is very much embedded on top of each other. So I can't talk about healthcare without thinking about early childhood care and what was received for the person because no matter whether you're homeless or housed, your early childhood care and attachment with your caregivers will predicate how you're were able to look after yourself. So for me, it's more so about people that aren't able to engage in a system that don't take into account where they're coming from, where they're at. . . If we can get system to understand where people are coming from, and have a system that meets people's needs, instead of the person having to fit into the system." Interviewee 8

“So it's well understood within the hospital system that when people have cancer, they should not be taking public transport and they need to be ferried from wherever they are to wherever they need to get for the treatment erm whereas it's not understood so much that same level of support might be required for someone to be able to walk from the hostel outside into the hospital for healthcare. They need some someone to support them to get to that point. Erm, and the culture very much in at times in the acute hospital setting can be very corporate. It can be very much inputs are patients and beds, outputs are bed days and what the cost [inauble]. . . that can be across medical systems, across the medical specialities so. And the culture sometimes can be that the leadership are kind of levels from middle up can be 'this person is in this bed for this length of days. They need to get out of that bed'. But blocks to them being able to go anywhere else are not seen. Because the understanding is not there because if you have a home, you can go home and convalesce for three weeks. If you're going into a hostel that's closed for 12 hours a day, that's not happening. So I think a lot of time it's when you're at that level of leadership and governance and you haven't had maybe that direct practice work that the healthcare professionals might have had, like, say for social work or OT or physio might have had direct contact with people.” – Interviewee 8

"So funding is allocated through what we would call a grant allocation and the grant allocation is under because we are Section 38 hospital, it's under a service level agreement. So there are a number of hospitals who are called Section 38. There's hospitals such as the [major Dublin hospital is a Section 38, because that's a pure voluntary hospital and then you've got HSE hospitals. So for us for instance, is that we get a funding allocation each year . . . there's a budget allocation to meet the existing level of services . . . And then if there's new services being developed, they go in through what we would call an estimates process. So every year does the estimates process where you submit a number of service developments that you want to get funded and the estimates process then goes into the HSE and then the HSE and the Department of Health go through it. And there's a cull as you can imagine of all the estimates and then just normally a few top priority programs then actually get funded".- Interviewee 10

“. . as you can appreciate, if you're running a health system, there's a number of different priorities that's all at the same time competing for funding. And there was a number of different priorities that would have been through the process an awful lot longer than a homeless one. And that that one actually got [funded]... And I think it's all about having the right advocates. And [name of doctor] would be very good, very good, strong advocate, Having the, you know, the ear of the minister, having a minister who understands the needs and having, you know, having a health system that can relate to it. And then often such things like covid or a crisis, actually can anticipate funding erm as well. So that that was one that actually was quite opportune, in relation that it did have the right conditions. And then there was a short-term funding made through the winter money. So winter money comes, but it's very short-term funding, you only get about six months funding but em because that was such a high demonstrated project . . So that's that's how new money comes into the system”. – Interviewee 10

"I think it's very, it's very ad hoc. And every funding that comes is sought for, for a long time. So the kind of lead is given by the voluntary sector, it grew up that way. And so each thing that emerges, it has to be pursued. So there isn't that kind of system at all. No, no, no, no, it's really it still is the Cinderella of government funding, you know, it's not long in existence". - Interviewee 11

"And even like for the so the funding model. So even on an abstract level, where does it where does the person start and end? The person starts and ends, in their home, in their family, in their community. They don't start and end erm marginalized, you know, and isolated as a, as a homeless person disconnected from everyone. They start and end in some home, in some family, in some community. And that's where the funding if you want to get really simple, should be targeted". – Interviewee 12
